# Prognostic Implications of Occult Nodal Metastases in Patients with Clinically N0 Primary Parotid Gland Cancer

**DOI:** 10.3390/medicina60121942

**Published:** 2024-11-25

**Authors:** Jooin Bang, Oh-Hyeong Lee, Geun-Jeon Kim, Dong-Il Sun, Sang-Yeon Kim

**Affiliations:** 1 Department of Otolaryngology-Head and Neck Surgery, Eunpyeong St. Mary’s Hospital, College of Medicine, The Catholic University of Korea, Seoul 06591, Republic of Korea; worldji@hanmail.net; 2 Department of Otolaryngology-Head and Neck Surgery, Seoul St. Mary’s Hospital, College of Medicine, The Catholic University of Korea, Seoul 06591, Republic of Korea; zzangyoh@gmail.com (O.-H.L.); emelenciana@naver.com (G.-J.K.); hnsdi@catholic.ac.kr (D.-I.S.)

**Keywords:** parotid neoplasm, lymphatic metastasis, lymph nodes, prognosis, disease-free survival

## Abstract

*Background and Objectives:* The role of occult nodal metastases in patients with parotid gland cancers remains unclear; such metastases are histologically diverse and exhibit unpredictable clinical courses. Here, we evaluated the prognostic utilities of such metastases, including metastases in the intraparenchymal lymph nodes (PARs). *Materials and Methods:* We retrospectively reviewed the medical charts of patients who underwent surgery to treat clinically N0 primary parotid gland cancers from 2000 to 2022. The primary outcome variables were 5-year overall survival (OS) and 5-year disease-free survival (DFS). We explored the effects of occult nodal metastases, including metastases in the PARs, especially in terms of the pathological T (pT) classification. *Results:* Among 74 patients, 48 (64.8%) were pT1/2 cases, and 26 (35.2%) were pT3/4 cases. Both perineural and lymphatic invasion were negatively associated with the 5-year DFS (hazard ratio [HR] = 3.533, 95% confidence interval [CI] = 1.325–9.421, *p* = 0.012; HR = 4.028, 95% CI = 1.497–10.839, *p* = 0.006, respectively). During pathological review, PAR metastases were observed in 12 patients (16.2%), and other occult metastases were present in 8 patients (10.8%). PAR metastases reduced the 5-year DFS in pT1/2 cases (87.2% vs. 22.2%, *p* = 0.001) but not in pT3/4 cases. *Conclusions:* PAR metastases significantly reduced the 5-year DFS in patients with clinically N0 primary parotid gland cancer. On subgroup analysis according to pT classification, this effect was significant among patients with early pT1/2 status but not patients with advanced pT3/4 status.

## 1. Introduction

Salivary gland cancers are relatively rare, constituting 1% to 3% of all head-and-neck cancers. However, such cancers pose significant challenges to otolaryngologists because their incidence has increased over the past several decades [1]. The histopathological subtypes of these cancers are diverse, their clinical behaviors are unpredictable, and their outcomes are variable. It is difficult to achieve a complete cure; the risk of recurrence is high [2,3]. A thorough understanding of the various factors that influence prognosis is essential. This understanding is particularly important in terms of parotid gland cancer, which is the most frequently affected salivary gland. Surgeons seek to optimize treatment and ensure that patients experience the best possible outcomes. Lymph node metastasis has extensive negative effects on prognosis, but the various factors independently prognostic on multivariate analysis differed among three recent reports [4,5,6]. In addition, the lymphatic drainage patterns of nodal metastases in patients with salivary gland cancers have not been fully elucidated; these drainage patterns are complex and thus difficult to define [7]. Although the optimal treatment for clinically N0 primary parotid gland cancers remains controversial, prophylactic neck dissection at levels I-III and/or Va is generally recommended. It has been reported that this approach ensures successful disease control [8].

Surgeons and other researchers who study parotid gland cancer have increasingly focused on cervical metastases in the PAR. These specialized lymph nodes lie within the parenchyma of the parotid gland; metastases in these nodes affect the prognoses and clinical outcomes of patients with parotid gland malignancies, significantly reducing recurrence-free survival [9,10]. The risk of distant metastasis increases as the numbers of metastatic PAR rise; this risk is greater than the risk associated with extranodal cancer extensions [11]. However, few studies have explored the impact of PAR metastases on prognosis; the evidence is limited. Here, we evaluated the clinical implications of occult lymph node metastases (including metastases in PARs) among patients with clinically N0 primary parotid gland cancer.

## 2. Materials and Methods

### 2.1. Patients

We retrospectively analyzed the medical records of 225 patients diagnosed with salivary gland cancers at Seoul St. Mary’s Hospital of the Catholic University of Korea between January 2000 and December 2022. We excluded patients with missing records, patients with parotid gland cancers that comprised metastatic lesions derived from other cancers, patients with submandibular or sublingual gland cancers, patients who did not undergo any form of surgery, patients who received neoadjuvant treatment prior to surgery, patients with a history of head-and-neck radiotherapy, and patients solely admitted for palliative care. Additionally, we excluded patients with malignancies that originated in the minor salivary glands; such cancers exhibit unique clinical characteristics. Finally, we included 74 patients. All pathological samples, including preoperative diagnostic biopsy samples and main surgically removed specimens, were examined by a single pathologist with many years of experience in the evaluation of head-and-neck malignancies. All cancers were categorized using criteria contained within the 8th edition of the American Joint Committee on Cancer guidelines.

### 2.2. Study Design

All patients with neck masses evident on initial physical examination underwent preoperative computed tomography and ultrasonographically guided fine-needle aspiration or core needle biopsy by a radiologist who specialized in such interventions. If a fine-needle aspiration biopsy specimen was insufficient to allow adequate histopathological evaluation, a core needle biopsy was conducted. However, if an initial core needle biopsy did not support a high suspicion of malignancy, we conducted an excisional biopsy with the patient under general anesthesia; we subsequently examined intraoperatively frozen specimens and scheduled appropriate treatment if the specimens showed positive results. The average time between the preoperative biopsy and the surgical treatment was approximately two weeks. We exclusively focused on patients with stage cN0, surgically resectable, primary, and parotid gland cancers. All such patients underwent upfront surgery with prophylactic neck dissection to assess occult lymph node status. Modified radical neck dissection was performed when the preoperative diagnostic biopsy result was negative, but an element of clinical uncertainty persisted, specifically when the preoperative assessment did not exclude histologically high-grade large tumors that may have invaded adjacent structures. Most patients underwent selective neck dissection of nodal levels I-III. After the collection of surgical specimens, we divided all patients into the following four or six categories. Patients who underwent selective neck dissection included individuals with metastases in PARs and nodes of the following levels: I, II, and III. Patients treated via modified radical neck dissection were divided into individuals with metastases in PARs and nodes of the following levels: I to V. Preoperative computed tomography or magnetic resonance imaging was used to determine the appropriate surgical approach. Based on the location and characteristics of the tumor, either partial or total parotidectomy was chosen by the principal surgeon. Superficial parotidectomy was used to remove tumors in the superficial lobe that were sufficiently distant from the facial nerve to allow preservation of that nerve. All adjuvant treatments were determined via multidisciplinary consultation involving experts in otolaryngology/head-and-neck surgery, pathology, radiology, radiation oncology, nuclear medicine, and oncology. If the pathological analysis revealed histologically high-grade tumors, close or positive resection margins, and/or perineural or lymphovascular invasion, we offered adjuvant treatment to affected patients after a careful explanation of the surgical plan; all patients then provided written informed consent. Additionally, adjuvant treatment was considered if the extent of surgery was inadequate because a tumor had been preoperatively misdiagnosed as benign, complete dissection could not be assured because the tumor adhered to adjacent structures, and/or tumor spillage had occurred during tumor excision. In these situations, patients received a careful explanation of the surgical plan and provided written informed consent. Seven patients underwent concurrent adjuvant chemoradiotherapy because their tumors were large, and microscopic invasion of adjacent structures was apparent on preoperative biopsy.

### 2.3. Outcome Assessments

The primary endpoint was the 5-year overall survival (OS): the time from surgery until death from any cause. The secondary endpoint was the 5-year disease-free survival (DFS): the time from surgery until death attributable to the recurrence of a major salivary gland cancer.

### 2.4. Statistical Analysis

All statistical analyses were conducted using SPSS ver. 25.0 software (IBM, Armonk, NY, USA). The chi-squared test or Fisher’s exact test was used to compare categorical variables; the t-test or Wilcoxon rank-sum test was used to compare continuous variables. We sought factors that independently influenced patient prognosis. The log-rank test was utilized to compare 5-year OS and 5-year DFS; the data are presented graphically in the form of Kaplan–Meier curves. The threshold for statistical significance was set to *p* < 0.05.

## 3. Results

### 3.1. Patient Characteristics

Patient demographic characteristics are listed in Table 1. The mean follow-up period was 92.5 ± 62.0 months. The mean patient age was 47.1 ± 15.9 years; there were 34 (45.9%) men and 40 (54.1%) women. Mucoepidermoid carcinomas were the most frequent tumors [30 cases (40.5%)], followed by adenoid cystic carcinomas (8 cases, 10.8%), salivary ductal carcinomas (7 cases, 9.5%), both acinic cell carcinomas and carcinomas ex pleomorphic adenomas (6 cases, 8.1% in total), squamous cell carcinomas (5 cases, 6.8%), both adenocarcinomas and cystadenocarcinomas (4 cases, 5.4% in total), epithelial myoepithelial carcinomas (2 cases, 4.3%), and 1 neuroendocrine carcinoma and 1 secretory carcinoma. Histopathologically, 38 tumors (48.7%) were high-grade, and 36 (51.4%) were low-grade. In terms of the pathological T (pT) classification, pT1 was the most common type [28 cases (37.8%)], followed by pT2 [20 cases (27.0%)], pT4 [15 cases (20.3%)], and pT3 [11 cases (14.9%)]. Fifty-nine patients (79.7%) underwent selective neck dissection, and fifteen patients (20.3%) underwent modified radical neck dissection. Fifty-eight patients remained free of disease (78.4%), but sixteen patients (21.6%) experienced recurrences. Among patients with recurrences, local recurrences and distant metastases were observed in seven and seven patients, respectively (43.8%); regional recurrences were detected in two patients (12.5%). The treatment modalities included surgery alone in 30 patients (40.5%) and surgery with adjuvant radiotherapy in 37 patients (50.0%). Seven patients (9.5%) underwent adjuvant concurrent chemoradiotherapy. The mean radiation dose (all patients who received radiotherapy) was 6223.1 ± 502.1 Gy.

### 3.2. Nodal Metastasis Locations in Patients with Clinically N0 Parotid Gland Cancers

**Figure 1:**
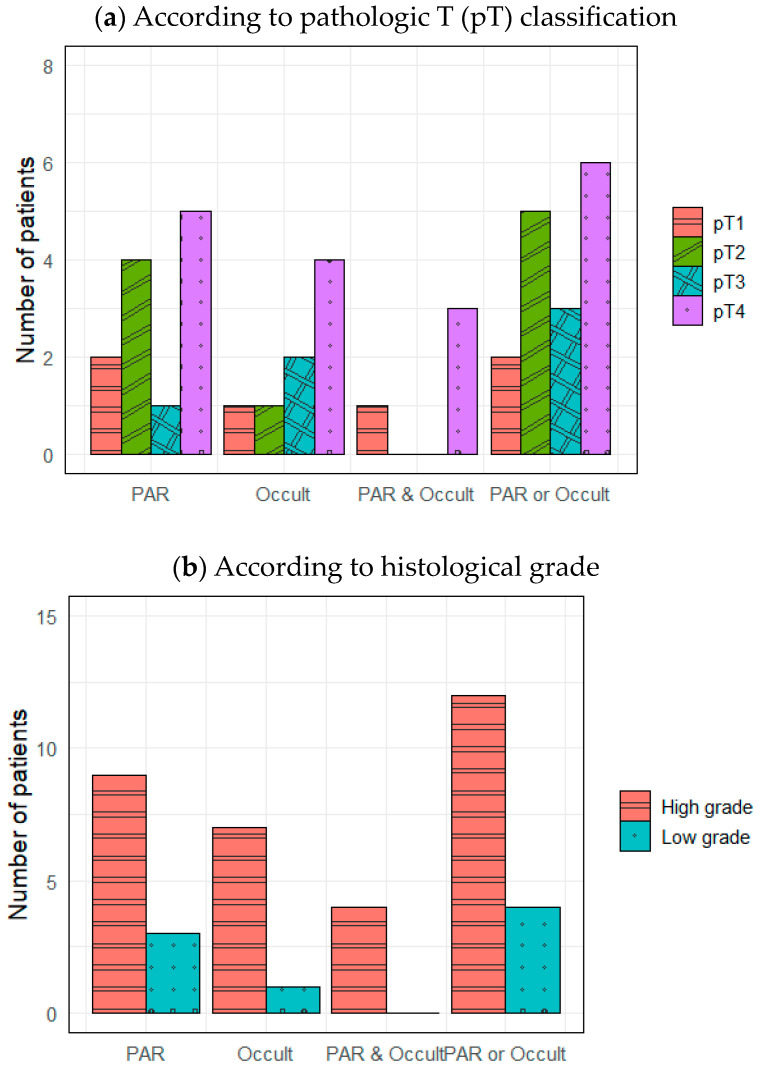
Distributions of lymph node metastases. PAR, intraparenchymal lymph node metastasis; Occult, occult neck lymph node metastasis except PAR.

Figure 1a,b show nodal metastasis locations based on pT classification and histological grade, respectively. The affected nodes included PARs, occult neck lymph nodes that were not PARs (i.e., “Occult” in Figure 1), both PARs and Occults, and at least one PAR or Occult. Both metastatic PARs and Occults were observed in patients with all pT classifications, but they were most common in pT4 patients (five with metastatic PARs and four with Occults) (Figure 1a). Both metastatic PARs and Occults were more frequent when the final histopathology was high-grade rather than low-grade (Figure 1b).

### 3.3. Risk Factors in Terms of 5-Year DFS

Table 2 lists prognostic factors for 5-year DFS. Women had better prognoses compared with men (hazard ratio [HR] = 0.346, 95% confidence interval [CI] = 0.120–0.996, *p* = 0.049). Among various histopathological factors, both perineural and lymphatic invasion significantly decreased the 5-year DFS (HR = 3.533, 95% CI = 1.325–9.421, *p* = 0.012; HR = 4.028, 95% CI = 1.497–10.839, *p* = 0.006, respectively). Other adverse histological features (e.g., close or positive margins and pericapsular and vascular invasion) also tended to worsen prognosis, but there were no significant differences between patients with and without these factors.

### 3.4. Effect of Metastatic Lymph Node Location on 5-Year DFS: Subgroup Analysis According to Patient pT Status

Table 3 lists the metastatic lymph node parameters analyzed in terms of their effects on 5-year DFS among patients with different pT classifications. Lymph node metastasis tended to decrease the 5-year DFS, but neither PAR metastases nor Occult metastases had statistically significant effects (both *p* > 0.05).

Table 4 lists the effects of metastatic lymph node location on 5-year DFS according to pT classification. In early pT1/2 cases, PAR metastases greatly reduced the 5-year DFS (HR = 7.107, 95% CI = 1.961–25.759, *p* = 0.003); the differences remained statistically significant when at least one metastatic PAR or metastatic Occult was present (HR = 5.219, 95% CI = 1.459–18.664, *p* = 0.011). Occult metastases alone tended to reduce the 5-year DFS, but this tendency was not statistically significant. However, PAR metastases did not significantly affect the 5-year DFS of pT3/4 cases (*p* = 0.995). Furthermore, Occult metastases did not significantly affect the 5-year DFS in this context (*p* = 0.486).

### 3.5. Impacts of Intraparenchymal Lymph Node Metastases on 5-Year DFS According to pT Status

**Figure 2:**
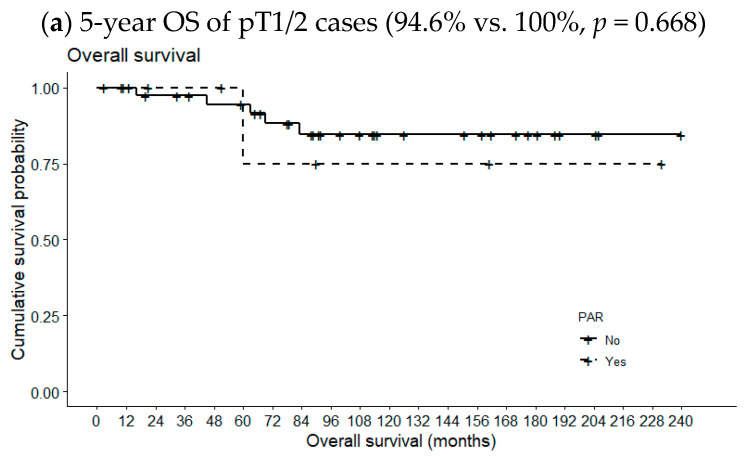

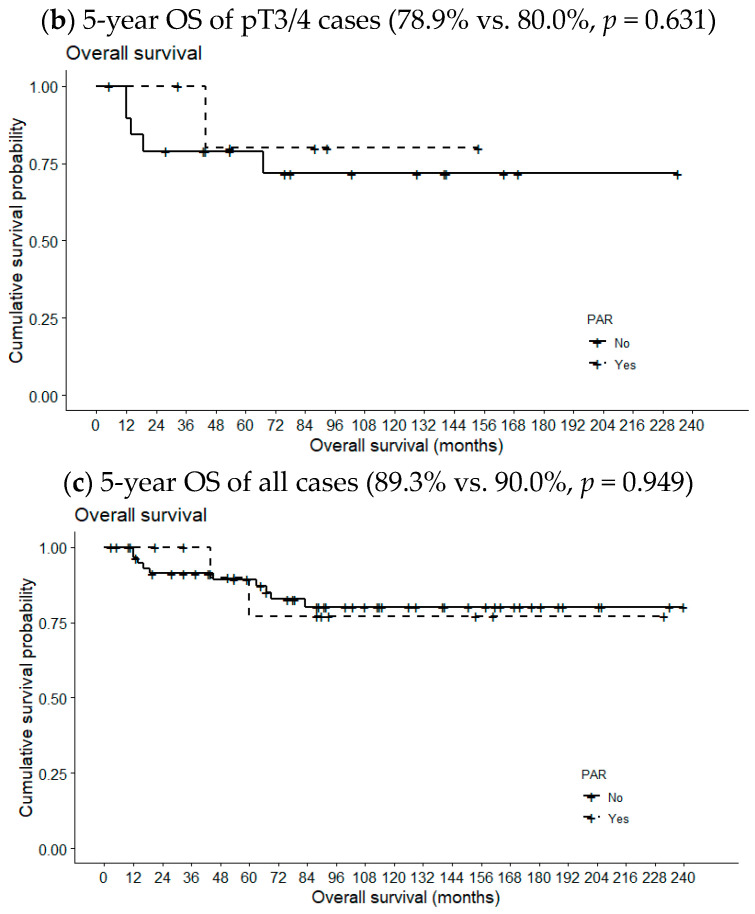
Effects of metastatic intraparenchymal lymph nodes on 5-year OS according to pT classification.

**Figure 3:**
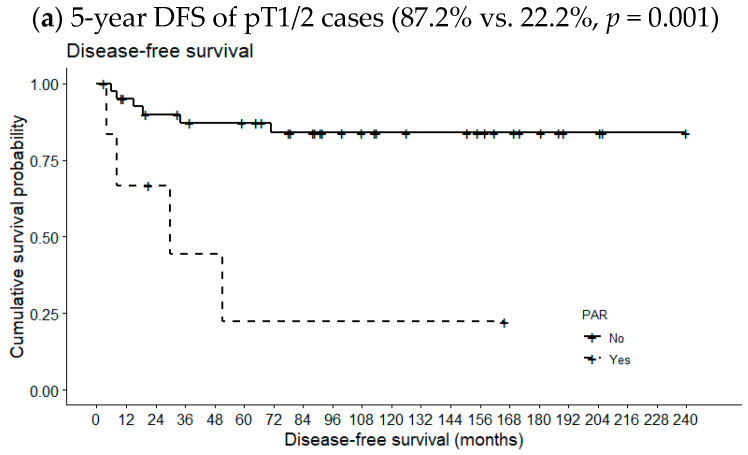

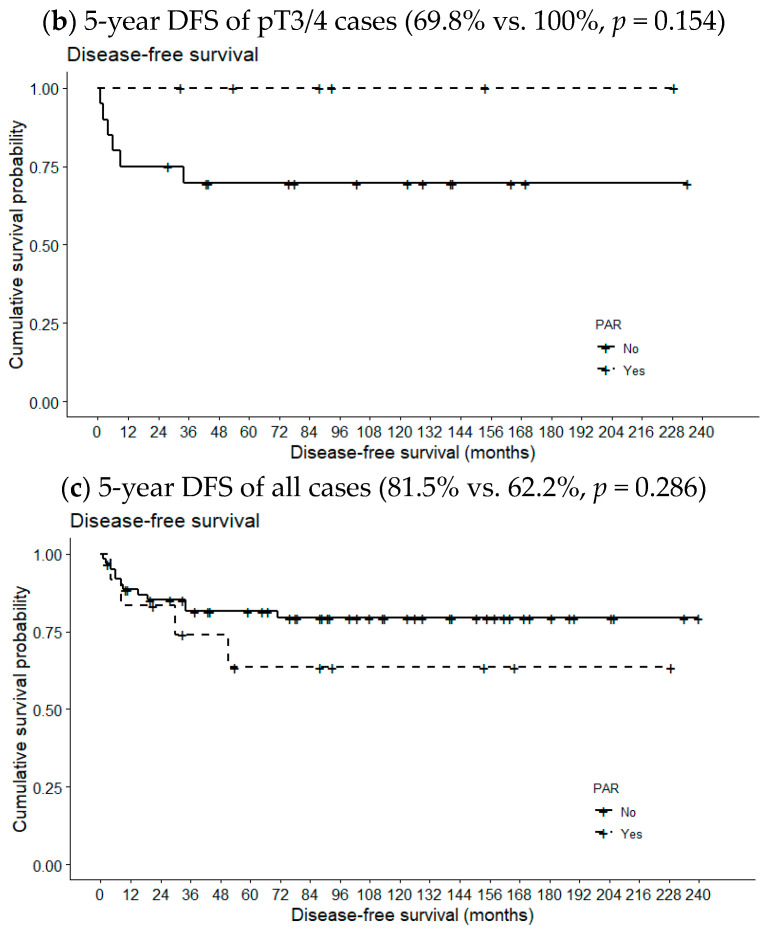
Effects of metastatic intraparenchymal lymph nodes on 5-year DFS according to pT classification.

Figure 2 compares 5-year OSs according to metastatic PAR status and pT classification. The 5-year OS of pT1/2 patients with metastatic PARs was slightly lower than the 5-year OS of patients lacking such lymph nodes (94.6% vs. 100%, *p* = 0.668). However, the opposite relationship was observed for pT3/4 cases (78.9% vs. 80.0%, *p* = 0.631); the differences were not statistically significant, even when the analysis included all pT classifications (89.3% vs. 90.0%, *p* = 0.949). However, the 5-year DFS of patients with metastatic PARs was significantly reduced in pT1/2 cases (87.2% vs. 22.2%, *p* = 0.001) but not in pT3/4 cases or all pT classifications (69.8% vs. 100%, *p* = 0.154 and 81.5 vs. 62.2%, *p* = 0.286, respectively) (Figure 3).

## 4. Discussion

The relationship between lymph node metastasis and prognosis in parotid gland cancer is a critical area of interest in head and neck oncology. Several studies have underscored the significance of nodal metastasis as a prognostic factor. A study by Meyer et al. found that an increased number of involved lymph nodes was associated with a significantly worse 5-year OS in patients with parotid gland cancer, showing 88% versus 37% whether the value of lymph node ratio (LNR) is less than 0.5 or not [12]. It emphasizes the adverse impact of a higher metastatic nodal burden on outcomes. Similarly, Elhusseiny et al. reported that increasing nodal burden over four involved nodes and over 33.3% LNR can predict poorer DFS with higher sensitivity (75.6%–81.1%) and specificity (72.8%–75.5%), suggesting the addition of metastatic nodal burden into the current staging system [13].

However, there is a debate over whether performing elective neck dissection (END) in clinically N0 primary parotid gland cancer can improve the prognosis or not. Stodulski et al. demonstrated that the proportion of metastases was higher than 20%, reaching up to 69% in levels I, II, and III, recommending END in the case of all pT3/4 as well as in pT1/2 with high-risk histology [14]. Whereas, a recently published retrospective study reported that END could not significantly increase the 3-year OS (69.7% in the END group vs. 69.4% in the observation group, *p* = 0.581), as well as the 3-year DFS (36.2% in the END group vs. 43.3% in the observation group, *p* = 0.728), even with advanced T3/4 parotid gland cancers [15]. In our study, we found that occult lymph node metastasis except PAR, i.e., Occult, was not associated with the 5-year OS and 5-year DFS, regardless of the pT classification (*p* = 0.875 in OS and *p* = 0.088 in DFS for all pT classifications). However, we found that when we included PAR with occult lymph nodes, it significantly decreased the 5-year DFS and the prognostic impact on 5-year DFS was more pronounced when the PAR was analyzed solely. This highlights the significance of PAR in occult lymph node metastasis and prompts us to analyze PAR by subgroup according to pT classification.

Embryologically, the submandibular and sublingual glands become encapsulated before the formation of the lymphatic system. While the PARs develop later in the parotid gland, these nodes reside within the parenchyma of the parotid gland [16]. In 1898, Neisse was the first to show that PARs typically lay in the inferior region of the superficial lobe of the parotid gland [16,17]. The importance of PAR involvement in terms of parotid gland cancer prognosis has become widely accepted. Lim et al. found that metastatic PARs increased the 3-year disease-specific mortality by more than 2-fold compared with non-metastatic PARs (75% vs. 34.2%, *p* = 0.0037) among patients with clinically negative nodal status, consistent with our findings [18]. In a large retrospective study, metastatic PARs were associated with a worse prognosis. The 10-year local control rate decreased as the number of metastatic PARs increased (the rates were 94% for patients lacking metastatic PARs, 56% for patients with no more than two metastatic PARs, and 22% for patients with more than two metastatic PARs) [19]. Although the prognostic significance of metastatic PARs has become widely recognized in recent years, any differences in terms of pT classification have received minimal attention. We found that the 5-year DFS was significantly higher in pT1/2 patients lacking metastatic PARs (71.4%) than in patients who had metastatic PARs (33.3%). We proceeded to analyze all pT classifications, but we found no prognostic significance of metastatic PARs in advanced pT3/4 patients. We do not seek to minimize the importance of metastatic PAR status in determining the prognosis of only early pT1/2 patients. Instead, we emphasize that early pT1/2 cancers may subsequently spread to the PARs; advanced pT3/4 cancers may exhibit more extensive metastatic involvement of other nodal levels, potentially diminishing the relative impact of metastatic PARs on prognosis.

We found that the frequency of occult metastases (i.e., lymph node metastases that did not include PAR metastases) linearly increased as the pT classification became more advanced. However, the metastatic PAR rate was lowest in pT3 patients. Because metastatic PARs may be present in early pT1/2 patients, intraglandular ultrasonographic evaluation of suspicious PARs is essential, regardless of pT classification. Patients with early pT1/2 cancer often only undergo partial parotidectomy. If the histology is low-grade, there is no tumor spillage during surgery, and perineural invasion is absent, the National Comprehensive Cancer Network guidelines indicate that radiotherapy may not be required. In some instances, surgeons become anxious that the disease might recur and cause psychological distress in the patient. However, our findings suggest that detailed preoperative evaluation of occult nodal metastases, particularly in PARs, in early pT1/2 patients reduces the risk of recurrence among patients for whom radiotherapy may not be required, ultimately enhancing the disease control rate.

This study had several limitations. First, any retrospective study in a single institution is inevitably associated with a risk of selection bias. Second, the statistical power and precision of our analyses were hindered by the small sample size. As mentioned above, salivary gland cancers are uncommon; it is difficult to accumulate large numbers of such patients. Third, the recently revised (2022) World Health Organization classification of salivary gland tumors was not considered when categorizing histopathological types. Despite these limitations, the present study offers some valuable insights. It serves as a foundation that will aid further research concerning how occult lymph node metastases affect the prognosis of clinically N0 primary parotid gland cancer. Our findings will aid surgeons who seek to ensure that patients are sufficiently cured to avoid disease recurrence.

## 5. Conclusions

The presence of metastatic PARs significantly reduced DFS, but such reductions were confined to patients with early pT1/2 clinically N0 primary parotid gland cancers. The DFSs of patients with advanced pT3/4 cancers and metastatic or nonmetastatic PARs were similar. Such findings suggest that detailed preoperative evaluation of metastatic PAR status for patients with early pT1/2 disease would reduce the risk of disease recurrence and improve disease control. To the best of our knowledge, this is the first report concerning the effects of metastatic PAR status according to pT classification. In practice, such evaluation can aid surgeons who must choose patient-tailored, clinical follow-up protocols that detect disease recurrence. A large, well-planned, multi-institutional study is required to validate our findings.

## Figures and Tables

**Table 1:** Patient demographics (*n* = 74).

Parameters	
Age, years	47.1 ± 15.9
Follow-up period, months	92.5 ± 62.0
Sex	
Male	34 (45.9%)
Female	40 (54.1%)
Pathologic type	
Mucoepidermoid carcinoma	30 (40.5%)
Acinic cell carcinoma	6 (8.1%)
Adenoid cystic carcinoma	8 (10.8%)
Carcinoma ex pleomorphic adenoma	6 (8.1%)
Salivary ductal carcinoma	7 (9.5%)
Squamous cell carcinoma	5 (6.8%)
Adenocarcinoma	4 (5.4%)
Cystadenocarcinoma	4 (5.4%)
Epithelial myoepithelial carcinoma	2 (2.7%)
Others (Small cell neuroendocrine carcinoma and secretory carcinoma, respectively)	2 (2.7%)
Grade	
High	38 (48.7%)
Low	36 (51.4%)
Pathological T (pT) classification	
pT1	28 (37.8%)
pT2	20 (27.0%)
pT3	11 (14.9%)
pT4	15 (20.3%)
Neck dissection	
Selective neck dissection	59 (79.7%)
Modified radical neck dissection	15 (20.3%)
Disease control status	
Disease-free	58 (78.4%)
Recurrence	16 (21.6%)
Local	7 (43.8%)
Regional	2 (12.5%)
Distant metastasis	7 (43.8%)
Postoperative treatment	
None	30 (40.5%)
Radiotherapy alone	37 (50.0%)
Concurrent chemoradiotherapy	7 (9.5%)
Radiation dose, Gy	6223.1 ± 502.1

Values are numbers (percentages) for categorical variables and means ± standard deviation for continuous variables.

**Table 2:** Factors affecting 5-year DFS.

Variables	HR (95% CI)	*p* Value
Age	1.004 (0.971–1.037)	0.831
Sex		0.049 *
Male	Reference	
Female	0.346 (0.120–0.996)	
Grade		0.059
High	Reference	
Low	0.360 (0.125–1.307)	
pT classification		0.770
pT1/2	Reference	
pT3/3	1.163 (0.423–3.201)	
Margin status		0.237
Clear	Reference	
Close or positive	1.980 (0.638–6.143)	
Pericapsular invasion		0.261
No	Reference	
Yes	1.836 (0.637–5.290)	
Perineural invasion		0.012 *
No	Reference	
Yes	3.533 (1.325–9.421)	
Vascular invasion		0.371
No	Reference	
Yes	1.967 (0.446–8.666)	
Lymphatic invasion		0.006 *
No	Reference	
Yes	4.028 (1.497–10.839)	

HR, hazard ratio; CI, confidence interval; pT, pathological T; *p* values are calculated using the Chi-square test or Fisher’s exact test for categorical variables and t-test or Wilcoxon rank sum test for continuous variables. * is for statistical significance.

**Table 3:** Effects of metastatic lymph node parameters on 5-year DFS according to pT classification.

Variables	HR (95% CI)	*p* Value
The presence of PAR		0.296
No	Reference	
Yes	1.831 (0.590–5.686)	
The presence of Occult		0.251
No	Reference	
Yes	2.088 (0.595–7.333)	
The presence of PAR or Occult		0.076
No	Reference	
Yes	2.503 (0.908–6.896)	

**Table 4:** Effects of metastatic lymph node location on 5-year DFS; subgroup analysis according to pT classification.

Variables	pT1/2		pT3/4	
	HR (95% CI)	*p* Value	HR (95% CI)	*p* Value
The presence of PAR		0.003 *		0.995
No	Reference		Reference	
Yes	7.107 (1.961–25.759)		0.000	
The presence of Occult		0.380		0.486
No	Reference		Reference	
Yes	2.525 (0.319–20.005)		1.830 (0.334–10.016)	
The presence of PAR or Occult		0.011 *		0.985
No	Reference		Reference	
Yes	5.219 (1.459–18.664)		0.984 (0.180–5.379)	

* is for statistical significance.

## Data Availability

The datasets used and/or analyzed during the current study are available from the corresponding author upon reasonable request.
